# Virus interference in CoViD-19

**DOI:** 10.6026/97320630018768

**Published:** 2022-09-30

**Authors:** Francesco Chiappelli, Lily Fotovat

**Affiliations:** 1 Dental Group of Sherman Oaks, CA 91403, USA

**Keywords:** Anti-viral immune response, major histocompatibility Complex (MHC), Interferon (IFN), IFN-inducible transmembrane (IFITM) proteins, RNA interference (RNAi), microRNA, Systemic Acute Respiratory Syndrome Corona virus2 (SARS-CoV2), Corona Virus Disease 2019 (CoViD-19), Receptor-Binding Domains (RBD), N-Terminal Domains (NTD) domain

## Abstract

Virus interference is one of the oldest concepts in immunology. Recent findings indicate that it may depend on the host's anti-viral cellular immune surveillance processes, as well as on sequence-specific gene silencing mechanism guided by double-stranded RNA. Other biological events, unrelated to some degree at least from immune-dependent IFN or RNA-dependent viral interference may be at play as well. We discuss these biological mechanisms in the context of of the Systemic Acute Respiratory Syndrome Corona virus2 (SARS-CoV2) virus responsible for Corona Virus Disease 2019 (CoViD-19).

## Background:

Virus interference is one of the oldest concepts in immunology, and may be dependent only in part on the host's major histocompatibility (MHC) genetic make-up. French philosopher Montaigne confirmed an observation recorded since antiquity that one disease could be, as he stated, cured or impeded by another in the mid-1500's. In the late 1700's, Edward Jenner's discovery of vaccination rested in part on the practice of variolation, which relied on the observation that exposure to cowpox could prevent the full manifestation of smallpox infection [1]. In the early 20th century, botanists observed that the yellow-mosaic tobacco virus could not replicate in plants previously infected with the common mosaic virus. The first experimental observations of virus interference in the animal kingdom were in 1935, which studied the differential tissue tropism of two strains of herpesvirus in rabbit. Flaviano Magrassi observed that rabbits infected with non-encephalitogenic strains of herpesvirus were resistant to infection by an encephalitogenic strain inoculated in the brain [2]. Two decades later, and following the WWII hiatus in experimental biological sciences, Isaacs and collaborators reported that, upon incubation of heat-inactivated influenza virus with fragments of live virus-infected chick chorio-allantoic membrane, a new factor was produced, which had the remarkable ability of interfering with virus replication in fresh pieces of chorio-allantoic membrane. The interfering factor could be replicable detected in the membranes after 3 h incubation, before being released into the incubation fluid. Because of its inherent ability to induce interference, the new factor was called interferon (IFN) [3].

Recent developments have established that IFN-independent, nucleic acid-based processes may also contribute to virus interference. As is the case in plants and insects, RNA interference (RNAi) is an efficient protective mechanism against viral infections in vertebrates. A variety of mammalian viruses encode suppressors of the RNAi pathway to block that antiviral mechanism. These suppressor molecules may come in the form of viral microRNAs or microRNA-like RNA molecules that are integrated within, and processed as part of the mammalian RNAi machinery. Host-encoded microRNAs can either silence or enhance intracellular levels of viral RNAs. In brief, interactions between the RNAi pathway and viral genomes modulate and regulate the life cycles of several viruses, and can interfere, by either blunting or boosting, the pathogenic signatures of the viral infectious agents [4].

These parameters are at play to differing extents in infection by the Systemic Acute Respiratory Syndrome Corona Virus2 (SARS-CoV2), which is responsible for the Corona Virus Disease 2019 (CoViD-19) pandemic. Alternatively, biologics-independent mechanisms, like the type of virus, titer, timing and sequence of exposure, also contribute to interfering with the SARS-CoV2 replication cycle and the onset and progression of CoViD-19.

## IFN Viral Interference:

Several diverse interferon proteins have now been identified to belong to the class of signaling proteins subsumed under the qualification of molecules used for communication between cells to trigger the protective defenses of the immune to help eradicate pathogens through cytokines. In brief, IFNs consist of a group of cytokines produced and released by host immune cells in response to the presence of certain viruses. The predominantly accepted mechanistic model states that a virus-infected cell can release IFNs that regulate the anti-viral immune defenses of nearby cells [5] (4). Today, over twenty distinct IFN genes and proteins have been identified, which contribute to immune clearance of viral infections and the regulation of the immune system. They are typically divided Type I IFNs, Type II IFNs, and Type III IFNs. Since all IFNs identified to date possess anti-viral abilities, they contribute, in a broad sense, to viral interference. In brief, Type I and II IFNs predominantly activate and regulate anti-viral cellular immunity. Type I and III IFNs are produced by virtually every cell types upon recognition of viral components, including viral nucleic acids and proteins. By contrast, Type II IFNs are induced strictly by immune killer cells (i.e., T or natural killer [NK]) by cytokines such as interleukin (IL)-12, and suppressed by IL10 [5]. All IFNs types function mechanistically by means of binding to specific receptors. Type I and II IFNs are the principal factors involved in viral interference by the primordial role they play in regulating and activating the anti-viral immune surveillance, whereas Type III IFNs appear to be relevant only in the case of certain viruses and fungi [5].In brief:

1. Type I IFNs are produced principally by cell populations of myeloid and fibroblastoid derivation and include IFN-α, IFN-β, IFN-ε, IFN-κ and IFN-ω. They bind to the two-chain IFN-α/β receptor (IFNa/bR1 and IFNa/bR2) on their specific target cells, and activate the signal transducer and activator of transcription (STAT) pathways for up-regulating expression of proteins that will prevent virus replication (i.e., production and replication of viral RNA and DNA). These properties have been exploited in the utilization of IFNs for treatment of viral disease, such as IFN-α to treat hepatitis B and C infections [5,6].

2. Type II IFNs, predominantly IFN-γ in humans is often designated as human immune interferon. Type II INFs, including IFN-γ, are produced and released by TH1 lymphocytes, and specifically CD8+ cytotoxic T cells in response to IL-12 stimulation. They bind specifically to the IFN-γ receptor (IFN-γR), which consists of two chains: the constitutive ligand-binding chain (α) IFN-γR1 also known as cluster of differentiation (CD)119, and the inducible trans-membrane Janus Kinase-STAT (JAK-STAT) and related STAT signal transducing IFN-γR2 (β) chain, the non-ligand-binding partner of the heterodimeric IFN-γR receptor [7,8].

3. Type III IFNs, including IFN-λ, are of more recent, and therefore to date less complete characterization. What is clear is that they play an important role in resistance to certain types of viruses and fungi by signaling through a receptor complex consisting of the β chain of the IL10 receptor (IL10R2, CRF2-4, CDw210B) and the constitutive α chain of the IL28 receptor (IL28R1, interferon lambda receptor 1, CRF2-12) [9-11].

In brief, anti-viral therapies have largely benefitted from the characterization of IFN's. Intramuscular or subcutaneous administration of IFNs have been found to be effective, either alone or supplementing pharmacological anti-virals or other modalities, for controlling a wide variety of virus-induced pathologies [5,11,12]. To be clear, all IFNs share the important properties of virus interference by being powerful antiviral agents, and to bring about this critical and timely activity by modulating anti-viral cellular immune functions of the immune system, including principally the activity of killer cells for removal and disposal of virally-infected cells. An important facet of IFN modulation of cellular immunity is their role in up-regulating major histocompatibility complex (MHC) molecules, those loci of the plasma membrane that are recognized as Self by the immune system. All IFNs induce Class I MHC, which is expressed on every cell type of the organism - a molecular signature, as it were, of the specific unicorn of the organism. Only Type II IFNs induce Class II MHC, which are only expressed on immune cell populations - their induction signifying an activation of the immune response [5,11].

## RNAi viral interference:

RNAi viral interference is a sequence-specific gene silencing mechanism guided by double-stranded RNA. Remarkably, the eukaryotic RNAi pathway is highly conserved, particularly between insects and mammals [13]. The introduction of small RNAi (sRNAi, or siRNA, or RNAsi) into eukaryotic cells efficiently blunts the viral cycle in vitro and in experimental animal systems, and is therefore hypothesized to be an effective therapeutic approach to inhibit virus replication in patients. Nonetheless, it is still unclear whether RNAi viral interference is equally effective across diverse families of DNA and RNA viruses, what dose and infection timing might be optimal, and whether or not there might be an MHC-derived host genome dependency upon RNAi virus interference effectiveness [13-14]. Mechanistically, most RNAi pathways across kingdoms and species share very fundamental steps, suggesting that it are a long ago-evolved process for ensuring the survival of an organism - plant, insect or vertebrate. Small RNAi regulate endogenous gene expression, protect the genome from invading transposons, and prevent the integration of viral nucleic acids at the pre- and post-transcription and epigenetic regulatory levels. All RNAi processes thus far identified share a common conserved effector complex that manifests as a protein - short single-stranded RNA complex. The protein of these complexes is invariably a member of the argonaute family [15]. Argonaute proteins are evolutionarily well-conserved peptidic components of the RNA-induced silencing complex that modulates gene silencing phenomenon, and hence RNA interference. Typically, small RNA molecules guide the argonaute to their specific targets through sequence complementarity and base pairing, which then results in mRNA cleaving by means of the argonaute endonuclease activity, and consequently gene expression silencing [16]. In the case of a viral infection, these argonaute-RNA complexes repress the transcription of viral genes, target mRNA for site-specific cleavage, and even block viral mRNA translation into proteins [15]. That certain argonaute protein, such as Argo4 seems to be particularly efficient in that modality with certain respiratory viruses in animal models suggests that they may be developed into effective anti-viral therapeutic strategies. Because small RNAi can effectively regulate the expression of a target gene by suppressing its mRNA transcription and translation in this regulatory pathway, it is possible and even likely that RNAi phenomena can juxtapose to create highly effective antiviral drugs. All is needed, theoretically at least, is a known sequence of the target viral protein. The clinical hypothesis then arises as to the possibility of utilizing RNAi application to suppress SARS-CoV-2, simply by either the early genomic characterization of the virus or the related SARS-CoV and MERS-CoV models [17]. Whether or not the RNAi database this developed would retain its effectiveness, and therefore its usefulness in light of the rapidly evolving variants and sub-variants, remains to be elucidated.

## Alternative viral interference:

Other biological processes, distinct, to some degree at least, from IFN or RNAi appear to mediate somewhat viral interference. IFN-inducible transmembrane (IFITM) proteins are cellular anti-viral proteins that restrict the replication of several, albeit not enveloped and non-enveloped viruses are involved. Members of the IFITM protein family are constitutively located in the plasma membrane and the intra-cellular endosomal membranes, at key entry portals for the viruses they block. In general, IFITM proteins effectively inhibit viral entry, possibly by altering the fluidity of cellular membranes [18]. It is possible and even likely that if IFITM proteins are represented in association with such ubiquitous receptors as ACE2, the portal of entry of SARS-CoV2, they may produce IFITM protein manifestations - i .e., altering cell membrane fluidity - with broad-base systemic consequences of potential long-term physiopathologic outcomes. One clinical hypothesis reasonably follows that states that Long Covid symptomatology are long-term manifestations of IFITM protein outcomes across systems and organs - or, otherwise stated, the result of IFITM protein-mediated interference against SARS-CoV2.

To be clear, IFITM proteins require carefully regulated post-translational modifications for their precise and accurate function. The process of post-translational modification is a condition sine qua non, a mechanism of timely and critical importance to shape, articulate and coordinate the action, structure and function of any and all proteins positively or negatively, whether or not they may be IFITM proteins, or involved in virus interferences at all. Case in point, IFITM3 is palmitoylated, and other related proteins endowed with antiviral properties, such as MxA, SAMHD1 and TRIM5α are SUMOylated (i.,e., covalent attachment of Small Ubiquitin-like Modifier [SUMO] protein), while BST2 is typically glycosylated (i.e., attachment of glycine [carbohydrate] to hydroxyl terminal). By contrast, viral proteins, including perhaps proteins encoded in the SARS-CoV2 genome, often evade restriction activity by inducing their ubiquitination and subsequent degradation [19].

Several other proteinic, other than IFITM, and non-proteinic cellular biochemical products contribute to blocking virus entry and blunting viral replication. As noted above, they include the IFN-inducible Mx1 protein (MxA) that acts as a powerful interfering protein against human influenza virus, the HIV-blocking SAM-domain and HD-domain containing protein1 (SAMHD1), and the retrovirus restriction factor Tripartite motif-containing protein 5alpha (TRIM5α), tethering (CD317, bone marrow stromal antigen2, BST2). They also include cholesterol 25-hydroxylase (CH25H), lymphocyte antigen 6E (LY6E), nuclear receptor co-activator protein 7 (NCOA7), interferon-γ-inducible lysosomal thiol reductase (GILT), HLA-DR-associated invariant gamma chain (CD74), and ADP-ribosylation factor GTPase activating protein (ARFGAP) with dual pleckstrin homology domain-containing protein 2 (ADAP2). They and many other that are just beginning to be uncovered form the first line of defense against virus infection, the initial front of viral interference [20]. Of note in this context is that CD317 has been reported to interfere strongly with SARS-CoV2, by impeding its release and shedding [21]. This research question is all the more important in light of the complexity of the emerging groups of variants and sub-variants of concern, as per CDC (Table 1 - see PDF) and of interest of this virus (Table 2 - see PDF) [22], and the observation that some, and perhaps most, or even all SARS-CoV2's can blunt IFN induction and IFN signaling, thus ensuring productive viral replication, shedding and infection within the host [23].

Several mechanisms may be involved in SARS-CoV2 escape from IFN vigilance, and altogether virus interference, from interactions at the molecular levels that range from preventing viral RNA recognition and inhibit the induction of IFN gene expression, to blocking the response to IFN treatment [23]. It is possible and even likely that the blunting effects of SARS-CoV2 on IFN contribute significantly not only to the recurrence of repeated serum-positivity in certain CoViD-19 patients, but also to the fast and apparently unrestricted spread of novel SARS-CoV2 variants and sub-variants. It follows that concerted investigative effort to better understand and characterize the modalities of escape of SARS-CoV2 from virus interference in general and IFN surveillance in particular is both timely and critical, lest CoViD-19 become the pathology "portal of entry, of unrelated viral diseases, such as monkeypox [24] or HIV/AIDS [24, 25]. Nonetheless, escape from immune surveillance and virus interference may not apply with respect to T cell-mediated immunity. Indeed, variants, T-cells recognize short amino acid linear peptides like spike receptor-binding domains (RBD) and N-Terminal domains (NTD's) domains, where most mutations occur in variants of concerns or interests. As a result, the T-cell responses remain largely intact against variants and sub-variants, including such aggressive variants as Omicron, despite reduced reactivity to antibodies or vaccines [26]. Case in point, a viral hierarchy has been proposed to explain, at least in part, escape from virus interference. Viral interference hierarchy may dependent upon the timing of exposure separating infection from once to the other virus, and, perhaps more importantly at the molecular level, independent from the antigenic similarities between the viruses. If that is indeed confirmed by data, then it signifies that one fundamental variable determining viral interferences may simply be the combination of sequential exposure. That is to say, the temporal hierarchy of infection, more than any other factor, might mediate and determine viral interference: the ability of one infecting virus to block or delay infection by another [27]. An experimental study utilizing a murine animal model, virus interference was introduced with influenza A-based interfering derived by a single central deletion from the full influence genome, which blunted disease onset by a second infection with a heterologous influenza b virus (IBV). "During the second infection, protection IBV was only slightly alleviated in mice that did not express a functional IFN-R1. Therefore, certain mice not having a functional IFN-R1 prevented them from contracting IBV. Similarly, this provided a certain layer of protection when a second infection of the pneumonia virus was introduced. Since the body was initially introduced to influenza, the second time the body was introduced to a strain of influenza or a similar respiratory virus, the body recognized its genetic sequence and was able to able to fight off infection. Having the blueprint for fighting the virus makes it easier for the body to fight off something similar in the future [28].

## Conclusions:

In conclusion, the concept of virus interference arose from early clinical observations that one virus may somehow prevent or reduce infection by another virus, and that, mechanistically; it might be mediated by a soluble factor, which was promptly named interferon (IFN). Research soon established that IFN consists of a large family of factors with related function, that certain members of that protein family have more potent anti-viral properties than others, and - as importantly - that virus interference could brought about by a large group of other proteins, some related to IFN (i.e., IFITM), and others clearly not. Third, and perhaps most relevant are the observations that virus interference need not be carried out at the level of proteins, but may, and does most certainly occur at the nucleic acid level. RNAi are timely and critical examples, particularly as concerted effort focuses on what the most efficient mechanism of virus interference might be for SARS-CoV2, the virus responsible for CoViD-19. Recent clinical findings confirm that timing of exposure may not be the only variable regulating the onset of viral interference, particularly in regards to patients with CoViD-19. The interactions between the D614G mutant SARS-CoV2 (i.e., replacement of l-aspartic acid, "D amino acid at position 614 of subunit 1 of the Spike protein with l-glycine, "G" amino acid, increasing the binding affinity to the ACE2 receptor and increasing the infectivity of the virus) with either influenza A(H1N1)pdm09 or type A2 respiratory syncytial virus (RSV) were tested in the nasal human airway epithelium, following either simultaneous or sequential (24 h apart) infection with these virus combinations. Viral replication kinetics of each virus by RT-qPCR at different post-infection times established that SARS-CoV2-D614G can effectively interfere with RSV-A2 but not with A(H1N1)pdm09 replication during simultaneous infection. Moreover, prior infection with SARS-CoV2-D614G reduced the replication kinetics of, that is to say, significantly interfered with infection by both RSV-A2 and A(H1N1)pdm09 respiratory viruses. By contrast, infection by SARS-CoV2D614G was markedly interfered by prior infection with A(H1N1)pdm09, but not RSV-A2. In other words, CoViD-19 may reduce the risk of influenza - an observation that seems to obtain support from some public health data [29], and exposure to the influenza virus A(H1N1)pdm09 may reduce subsequent infection with certain SARS-CoV2 variants. The mechanism involved in the viral interference between SARS-CoV-2 and A(H1N1)pdm09 appeared to be IFN-dependent [30]. Taken together, these lines of evidence confirm and in part explain the observations of a recent systematic review of close to 10,000 patients that established that co-infection with SARS-CoV2 and influenza virus had no effect on overall mortality, and indeed lowered risk for critical clinical outcomes [31]. A second independent meta-analysis of 23 peer-reviewed homogeneous research reports involving over one million subjects established that the tetravalent anti-influenza vaccine raised against two influenza A viruses and two influenza B viruses lowered the risk ratio for CoViD-19 (RR=0.74, 95% CI=0.65, 0.84), and actually improved clinically outcomes [31].

## References

[1] Chiappelli F (2023). Chapter 1 In GV V: Virus Vaccines..

[2] Magrassi F (1935). Zeitschrift fur Hygiene und Infektionskrankheiten..

[3] Isaacs A (1957). Proc R Soc London Ser B - Biol Sci.

[4] Schudz S, Sarnow P (2006). Virology..

[5] Parkin J, Cohen B (2001). Lancet..

[6] Levy DE (2011). Curr Opin Virol..

[7] Luster AD (1988). J Biol Chem..

[8] Kotenko SV (1995). J Biol Chem..

[9] Hermant P, Michiels T (2014). J Innate Immun..

[10] Espinosa V (2017). Sci Immunol..

[11] Borden EC (2007). Nat Rev Drug Discov..

[12] Shi LZ, Bonner JA (2021). Axis. Int J Mol Sci..

[13] van Rij RP, Andino R (2006). Trends Biotechnol..

[14] Ding SW (2018). Curr Opin Immunol..

[15] Sioud M (2021). Methods Mol Biol..

[16] Jonas S, Izaurralde E (2015). Nat Rev Genet..

[17] Pashkov EA (2021). Vopr Virusol..

[18] Smith S (2014). Curr Opin Virol..

[19] Chamontin C (2021). Viruses..

[20] Majdoul S, Compton AA (2022). Nat Rev Immunol..

[21] Martin-Sancho L (2021). Mol Cell..

[22] https://pubmed.ncbi.nlm.nih.gov/34033342/.

[23] Beyer DK, Forero A (2022). J Mol Biol..

[24] Farahat RA (2022). Ann Clin Microbiol Antimicrob..

[25] Nolasco S (2022). J Infect..

[26] Wherry EJ, Barouch DH (2022). Science..

[27] Laurie KL (2015). J Infect Dis..

[28] Koutsakos M (2021). Nat Rev Microbiol..

[29] Fage C (2022). Viruses..

[30] Guan Z (2021). Front Public Health..

[31] Su W (2022). Am J Prev Med..

